# Suppressing Halide Segregation in Wide‐Bandgap Perovskite Absorbers by Transamination of Formamidinium

**DOI:** 10.1002/cphc.202500022

**Published:** 2025-04-17

**Authors:** Georgios Loukeris, Clemens Baretzky, Dmitry Bogachuk, Audrey Elizabeth Gillen, Bowen Yang, Jiajia Suo, Waldemar Kaiser, Edoardo Mosconi, Filippo De Angelis, Gerrit Boschloo, Andreas Walter Bett, Uli Würfel, Markus Kohlstädt

**Affiliations:** ^1^ Fraunhofer Institute für Solar Energy Systems ISE 79110 Freiburg im Breisgau Germany; ^2^ Materials Research Center FMF University of Freiburg 79104 Freiburg im Breisgau Germany; ^3^ Institute of Physics University of Freiburg 79104 Freiburg im Breisgau Germany; ^4^ Department of Chemistry ‐ Ångström Laboratory 75237 Uppsala Sweden; ^5^ Computational Laboratory for Hybrid/Organic Photovoltaics (CLHYO) Instituto CNR di Scienze e Tecnologie Chimiche “Giulio Natta” (CNR‐SCITEC) 06123 Perugia Italy; ^6^ Department of Chemistry, Biology and Biotechnology University of Perugia and INSTM 06123 Perugia Italy; ^7^ SKKU Institute of Energy Science and Technology (SIEST) Sungkyunkwan University Suwon 440‐746 Korea; ^8^ Present address: Solarlab Aiko Europe GmbH Berliner Allee 29 79110 Freiburg Germany; ^9^ Present address: Department of Physics, TUM School of Natural Sciences Technical University of Munich 85748 Garching Germany

**Keywords:** bulk passivation, density functional theory calculations, long‐term illumination, photostability, wide‐band gap perovskite

## Abstract

All‐perovskite tandem solar cells are emerging at a fast rate because of their potential to exceed efficiencies of Si‐perovskite tandems, in combination with faster manufacturing, lower cost, and the ability to be processed on flexible substrates. Mixing halides is a key to achieve wide‐bandgap absorbers, which however suffer from halide segregation under illumination, resulting in lowering of the bandgap. To tackle this problem, butylamine (BA) has been added to the perovskite precursor solution and is found to react with the formamidinium (FA) cation, producing N‐butylformamidinium (BuFA^+^), which accumulates at the perovskite surface and grain boundaries. The creation of the BuFA cation results in suppressed halide segregation and improved crystallization. Density functional theory calculations propose the reduction of halide defect formation upon the addition of BA, being a key to stabilize mixed‐halide perovskites. Lastly, we observe a more stable performance of single junction p–i–n perovskite solar cells with the addition of BA under constant illumination at 65 °C.

## Graphical abstract

Photostable wide‐bandgap (1.77 eV) perovskite solar cells were created by adding butylamine (BA) to the precursor solution. Density functional theory calculations predicted that BA reacts with the precursor forming the novel cation N‐butylformamidinium (BuFA^+^), which is proven by ^1^H nuclear magnetic resonance spectroscopy. From photoluminescence measurements, the BA addition is shown to suppress the negative halide segregation effect, increasing the stability of passivated solar cells.

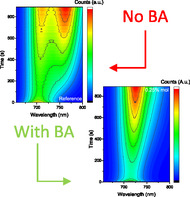

## 1 Introduction

Metal halide perovskite (MHP) photovoltaics have been rapidly developed over the course of recent years, leading to a tremendous increase in single junction efficiencies from 3.9% to 26%.^[^ ^1^ ^]^ Besides high absorption coefficient, long charge carrier diffusion length, small exciton binding energy, and high charge carrier mobility, MHPs provide the additional ability of bandgap energy tunability by varying the absorber's stoichiometry.^[^ ^2^, ^3^, ^4^, ^5^, ^6^, ^7^ ^]^ The bandgap tunability makes this material class uniquely prominent in all‐perovskite tandem applications, by combining two MHP photoabsorbers with different bandgaps. In a typical state‐of‐the‐art all‐perovskite tandem device, a narrow bandgap (NBG) absorber is composed of a Pb–Sn mixed perovskite with a Pb:Sn ratio of 50:50 to achieve a bandgap of about 1.22 eV, while for the wide‐bandgap (WBG) perovskite absorber a halide ratio (I:Br) of 60:40 is used to reach a bandgap energies of ≈1.75 eV.^[^ ^8^, ^9^, ^10^, ^11^, ^12^, ^13^ ^]^

However, mixed‐halide perovskites suffer from halide segregation, also known as the Hoke effect, under operating conditions, illumination, heat, and electrical biasing, that can be reversible in the dark.^[^ ^14^, ^15^, ^16^, ^17^, ^18^ ^]^ It is understood that, due to a low energetic barrier for ion migration, I^−^ ions tend to migrate and accumulate together leading to the formation of I‐rich domains.^[^ ^15^, ^19^, ^20^, ^21^ ^]^ Particular focus has been recently put on the role of iodide oxidation as a driving force for halide segregation, likely being accompanied by iodine release and iodide vacancy formation.^[^ ^22^, ^23^, ^24^ ^]^ Concomitantly, regions with high densities of iodide vacancies and incorporated Br^−^ become Br‐rich domains. This spatial inhomogeneity in the halide ratio leads to a spatial variation in the bandgap, resulting in an open‐circuit voltage (*V*_OC_) deficit and poor stability. Especially in tandem applications, halide segregation raises substantial concerns, since the decrease in bandgap of the WBG absorber results in deviations from the optimal combination of bandgap energies between WBG and NBG absorbers, severely decreasing device efficiency.

To tackle these photoinduced stability problems, the scientific community focused on fine‐tuning the composition of the WBG perovskite precursor by exchanging the methylammonium (MA) for the cesium (Cs) cation, incorporating rubidium iodide (RbI) or bulky cations, such as dimethylamine (DMA) stoichiometrically, optimizing interfaces and interlayers.^[^ ^18^, ^25^, ^26^, ^27^, ^28^ ^]^

In this work, we present a novel method to improve the phase stability of mixed‐halide perovskites by the addition of butylamine (BA) to the WBG perovskite precursor solution. We observe a transamination reaction between the amine and the formamidinium (FA) cation, creating N‐butylformamidinium (BuFA^+^), a bulky monovalent organic cation that improves film morphology, optoelectronic quality, and stability. We found that BuFA^+^ localizes at the perovskite surface and grain boundaries, where it causes a reduction in iodide defect formation as observed from density functional theory (DFT) calculations. Finally, we demonstrate that such incorporation of BA into the perovskite solution represents a facile method to effectively suppress halide segregation for an extended period of time, enhancing the power conversion efficiency of perovskite solar cells and increasing the stability of devices in both inert atmosphere and under continuous illumination in ambient.

## 2 Results and Discussion

**Figure 1:**
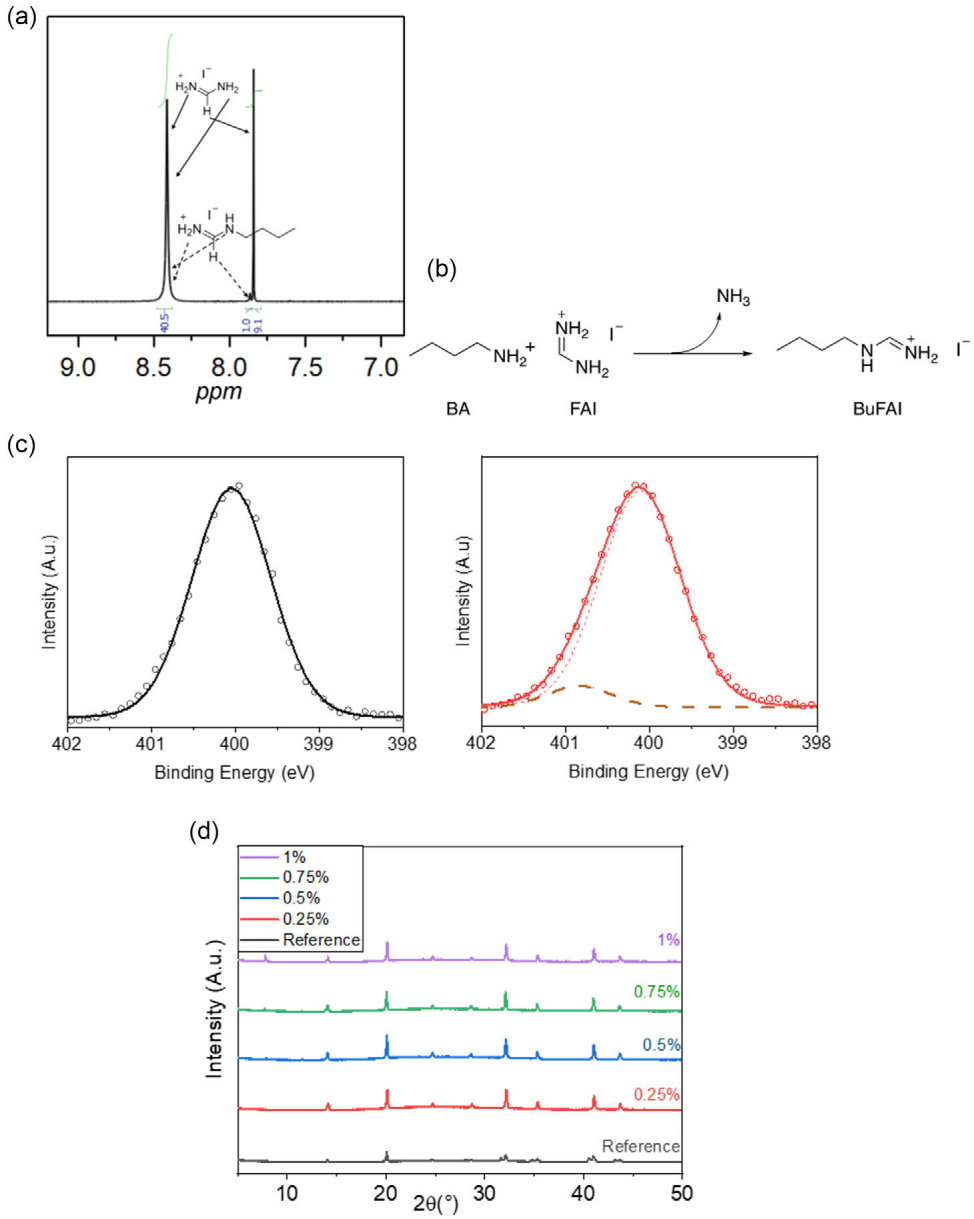
a) ^1^H NMR spectrum of a mixed solution of FAI and BA (ratio 10:1) in isopropanol. b) Scheme of the transamination reaction between BA and FAI. c) XPS N1s spectra of undoped (left) and BA‐doped (right) perovskite films on glass. d) Small‐angle X‐ray diffraction (XRD) on BA‐doped perovskite films with varying BA concatenation.

Recently it has been demonstrated that aliphatic amines react with FA by a nucleophilic attack of the imine bond, leading to a release of ammonia and the formation of bulky FA‐based cation with an alkyl chain bonded to it.^[^ ^29^, ^30^ ^]^ Such cations are used to passivate surfaces and grain boundaries and further form low‐dimensional perovskites, which are known to passivate surface defects, reduce nonradiative recombination, and enhance device stability.^[^ ^31^, ^32^ ^]^ To investigate the reaction between BA and FAI in the precursor solution, ^1^H nuclear magnetic resonance (^1^H NMR) measurements were carried out. BA, FAI, and the mixture (ratio of BA to FAI is 10:1) were dissolved in DMSO‐d6, respectively. The full spectra of FAI, BA, and the mixture of FAI + BA in addition to the highlighted area of FAI + BA interaction are presented in Figure S1a,b,c, Supporting Information. As shown in the spectrum of FAI solution, two peaks were observed at 8.75 and 7.86 ppm, corresponding to the four active protons of the amino group, and the proton connecting with the carbon atom, respectively. After adding BA into the FAI solution, BA will nucleophilic attack FA^+^ to form N‐butylformamidinium (BuFA^+^) by releasing ammonia. Thus, a new peak can be observed at 7.88 ppm, corresponding to a proton connecting with the imine carbon atom in BuFA^+^ as shown in **Figure** **1**a. The newly formed active protons were mixed with FA^+^, which agrees with the integrations of different peaks as depicted in Figure 1a. The reaction scheme is specified in Figure 1b. DFT calculations suggest the favorable formation of BuFA^+^ with a computed energy difference of ΔE = −0.16 eV between the BuFA^+^ product and the reactants FA^+^ and BA, according to the reaction scheme in Figure 1b (see Supporting Information for computational details). Additionally, from X‐ray photoelectron spectroscopy (XPS) measurements (Figure 1c) (full spectra can be found at the Figure S2a,b, Supporting Information), it can be seen that the nitrogen contained in FAI reacts with BA, indicated by a new peak that is observed at higher binding energies (401 eV). This finding further verifies the reaction of the BA with the perovskite absorber.^[^ ^33^ ^]^

Due to their bulky nature, BuFA^+^ cations cannot enter the lead–halide cages in the 3D perovskite lattice. Thus, BuFA^+^ should reside on surfaces and grain boundaries, or even form quasi‐2D phases, the amount of which must be increasing with increasing BA concentration. To understand where BuFA^+^ is located, we prepared perovskite films produced with various amounts of BA and performed grazing incidence X‐ray diffraction measurements. As shown in Figure 1d, a distinct peak at 7.8° appears at 0.75 mol% and increases in intensity with a higher concentration of BA in the precursor solution. We may assign this peak to a 2D phase, while at lower concentrations BuFA^+^ is expected to reside on surfaces and grain boundaries. This finding comes in perfect agreement with recent reports on FA containing perovskites.^[^ ^34^, ^35^ ^]^

**Figure 2:**
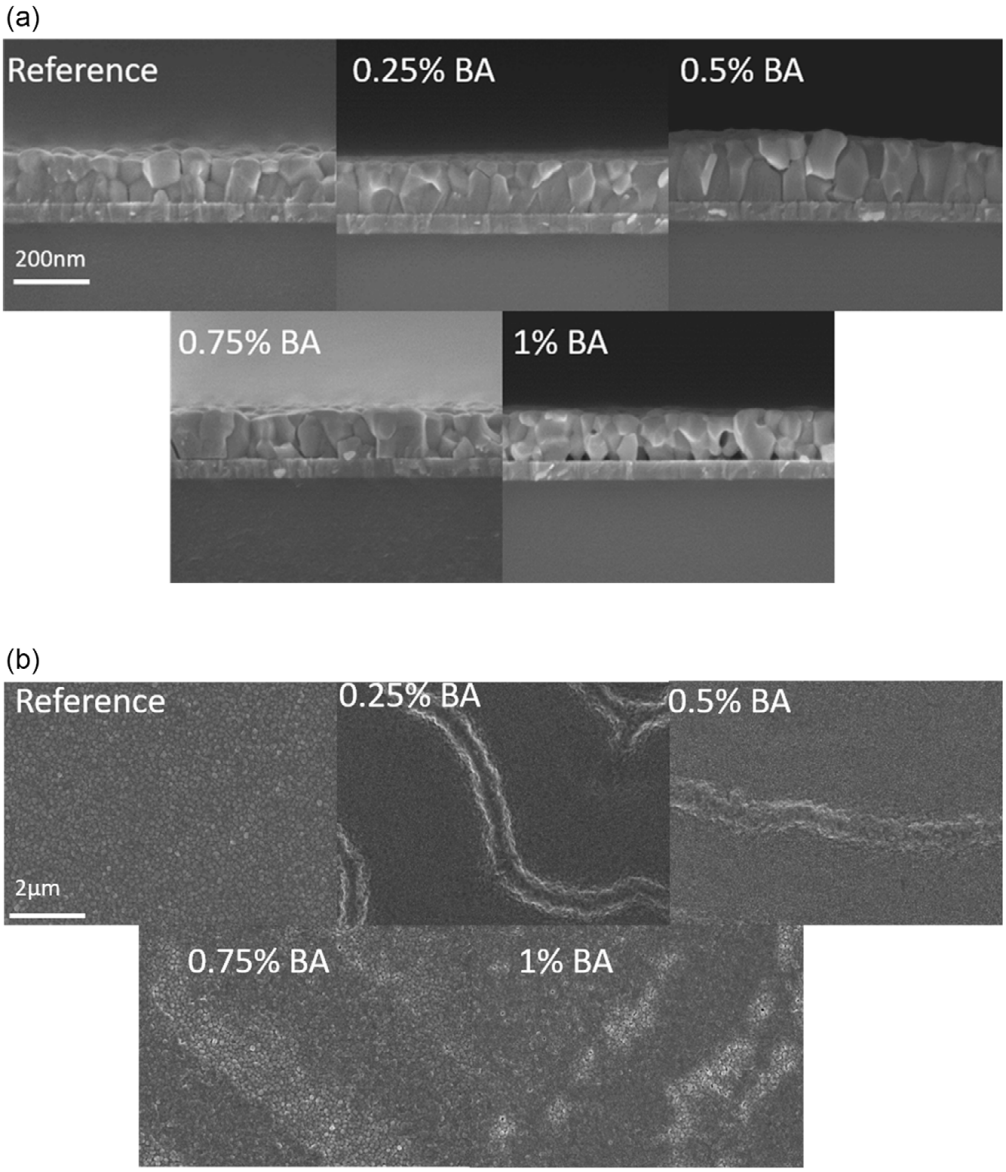
SEM images of films with varying BA concentration. a) Cross‐sectional SEM images of WBG perovskite thin‐films with varying additive (BA) concentration. The concentration variation corresponds to %mol of lead. b) Top‐view SEM images of WBG perovskite thin‐films with varying additive (BA) concentration. The concentration variation corresponds to %mol of lead.

At a later stage, scanning electron microscopy (SEM) measurements of perovskite films with varying BA concentrations were performed in order to identify its role in the crystallization. In **Figure** **2**a, cross‐sectional SEM images show that by increasing the BA concentration, bigger perovskite grains are formed, and single grains can be identified throughout the whole thickness of the film. Unfortunately, the grains tend to be misaligned, while at the same time cracks and gaps between the substrate and the perovskite's surface are formed at higher BA concentrations of ≥0.5%. This effect is correlated with the degassing of volatile molecules, such as Ammonia, which is a by‐product of the interaction between FA and BA as shown in Figure 1b.^[^ ^36^, ^37^ ^]^ The mechanism behind the optimal crystallization is the matching of the NH_3_ degassing rate to the perovskite nucleation rate. This effect takes place when 0.25% BA is added to the absorber. As the concentration of the BA increases, more NH_3_ is produced, leading to unmatched degassing to nucleation rates. Moreover, Figure 2b shows large‐scale top view images of perovskite films with varying BA concentrations.^[^ ^38^ ^]^ From the surface morphology, ripples can be seen, which become more prominent for low amounts of the additive, which diminish for the highest additive concentration and are not present for the reference device. It is known from the literature that WBG perovskite devices featuring the ripples perform better than those without, due to overall stress relief because mechanical stress acts as a force to induce degradation.^[^ ^39^, ^40^ ^]^ Additionally, increased layer thickness causes an increase of the photogenerated current; and thus, short‐circuit current density (*J*_SC_) and changing internal reflection properties that further boost *J*_SC_ making a wavy perovskite surface a desired feature for this system. Lastly, white spots which correlate with nonconverted PbI_2_ are present in films with high BA concentration and the reference.^[^ ^41^ ^]^ Overall, optimal morphology is obtained for the 0.25% mol concentration of BA, since no cracks and voids are formed, the grains are well aligned, the ripples are present, and no unconverted PbI_2_ is observed. This result suggests that the 0.25% mol concentration of BA would be optimal to create perovskite layers with better crystallinity, enhancing overall solar cell performance.

**Figure 3:**
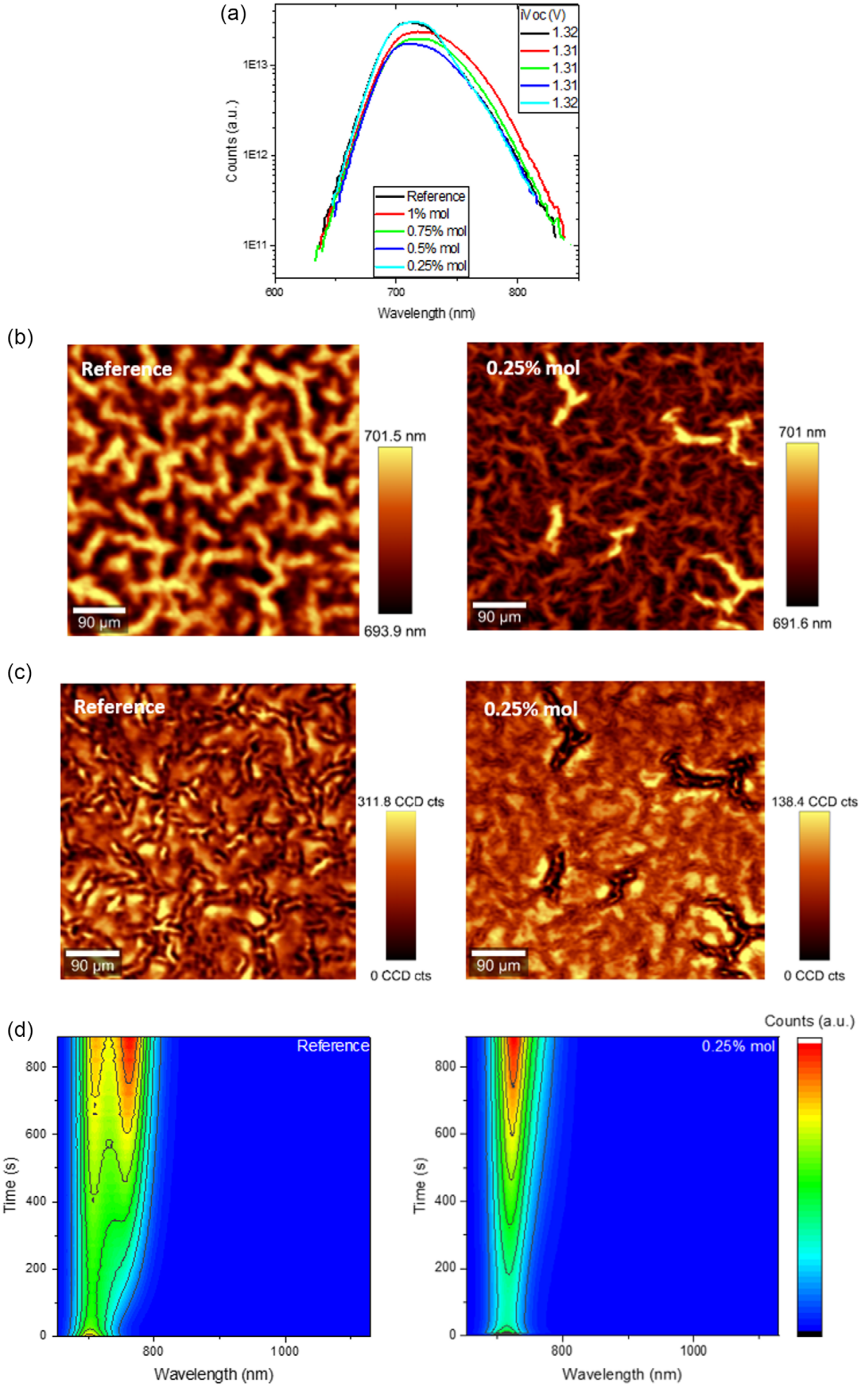
Photoluminescence measurements performed to identify electrical and optical absorber properties. a) PLQY measurements of thin‐films with varying BA concentrations. The legend corresponds to different concentrations and the corresponding i*V*_OC_. b) Wavelength resolved μ‐PL mapping comparing the nonpassivated with the 0.25% BA film. c) Intensity resolved μ‐PL mapping comparing the nonpassivated with the 0.25% BA film. d) Steady‐state PL results comparing the time evolution of the emission spectrum.

To understand the effect of BA treatment on halide segregation, WBG perovskites with varying amounts of BA were coated on bare glass and investigated by photoluminescence techniques. As shown in **Figure** **3**a, the addition of BA does not show an increase in the implied *V*_OC_ (i*V*_OC_), which would suggest that solar cells created with this additive do not provide higher *V*_OC_. In conjunction with this finding, photoluminescence measurements from all samples do not show differences in the maximum counts of emission.

Moreover, micro‐photoluminescence (μ‐PL) measurements were performed on perovskite films on glass substrates with varying BA concentrations to identify the homogeneity of the emission spectrum (Figure 3b,c). In comparison to the reference films, we observed a pronounced spectral emission homogeneity for the 0.25% BA‐treated film (spatially resolved photoluminescence spectra for all variations can be seen in Figure S3a, Supporting Information). From wavelength‐dependent mapping, we can see that the BA‐treated film shows a more homogeneous emission spectrum than the reference, while from intensity‐dependent mapping, the same trend is shown. This finding comes in good agreement with the enhanced crystallinity in the BA‐treated films, as shown in SEM images in Figure 2. To gain more insights on the morphology of the perovskite surface, microscopy measurements were conducted on samples with varying BA concentrations. From, Figure S4, Supporting Information shows that all samples contain striations, as shown in Figure 3b,c and Figure S3, Supporting Information, which points to the direction that this is a universal WBG absorber effect.^[^ ^40^ ^]^ The maximum intensity collected is in the same order of magnitude for all variations, which comes in good agreement with photoluminescence quantum yield (PLQY) measurements. Furthermore, steady‐state PL measurements were conducted for all samples, as shown in Figure 3d. After 15 min of continuous illumination, the reference device has developed a second segregated peak at ≈760 nm, while the passivated devices with 0.25% mol BA concentration show a negligible shift (reflection–transmission–absorption measurements were conducted to verify the bandgap value of the reference and BA‐treated perovskite films as shown in Figure S5, Supporting Information). Additionally, as shown in Figure S6, Supporting Information, all BA‐treated films show the halide segregation effect in a less pronounced extent than the nonpassivated device. This suggests a highly beneficial role of the BuFA cation on the surface and grain boundaries in stabilizing the mixed‐halide perovskite phase. Lastly, transient photoluminescence measurements were performed of reference and BA‐treated perovskite films (can be seen in Figure S7, Supporting Information). The tr‐PL signal at longer timescales corresponds to charge carriers that are trapped in shallow trap states in the bulk and eventually released again; thus, exhibiting higher lifetimes. As shown, for the lowest amounts of BA in the perovskite absorber, the intensity of the tr‐PL signal collected from these energy states is lower, meaning that less trap states are present in these films. To quantify this finding, there is an order of magnitude count difference between the lowest BA concentrations to all other variations. The radiative recombination rate of solar cells is equal to the product of the charge carrier densities. Additionally, one order of magnitude difference in the emission counts means that there a ten‐fold less electrons and holes trapped in these shallow trap states.

Next, DFT calculations were performed to rationalize the origin of suppressed halide segregation in the presence of BuFA cations. As noted earlier, the BuFA cations reside on the grain surfaces and boundaries. At low concentrations of BA in solution, no 2D phase is forming. Thus, we consider the role of BuFA^+^ on the 3D surface of mixed‐halide perovskites. For simplicity, we model a mixed‐halide perovskite slab using FAPb (*I*_0.67_ *Br*_0.33_)_3_ as our model system with different terminations. Particularly, we compare the role of FA^+^ and BuFA cations on the formation of halide defects at the perovskite surface, see Figure S8, Supporting Information for representative surface models and Supporting Information for computational details. Recent studies on halide segregation strongly emphasize on iodide oxidation, resulting in iodine release upon illumination as the main cause driving phase segregation.^[^ ^24^, ^42^ ^]^ To model this process, we consider the reaction
(1)

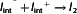




**Figure 4:**
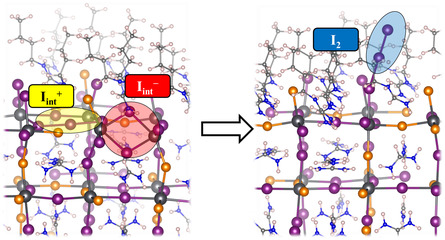
Atomistic surface model of a BuFAX‐terminated FAPb(*I*_0.67_ *Br*_0.33_)_3_ perovskite showing the formation of molecular iodine (right) starting from (left) oxidized iodide interstitials (*I*_int_^+^/*I*_int_^−^) under illumination.

This reaction first requires the formation of negative and positive iodide interstitial defects (*I*_int_^−^ and *I*_int_^+^, respectively) at the perovskite surface with subsequent formation of molecular iodine. On the pure random (X) (FAX)‐terminated surface, DFT calculations predict the formation energy for an *I*_*i*nt_^−^/*I*_int_^+^ pair to be 0.89 eV and an energy of −0.69 eV for reaction (1), see **Table** **1** and **Figure** **4**. In contrast, the BuFAX‐terminated surface shows an increased formation energy for the *I*_int_^−^/*I*_int_^+^ pair of 1.20 eV, lowering the density of such oxidized iodide defects. When being present, the release of I_2_ at the BuFAI‐terminated surface is still highly favorable, showing energies of −1.35 eV for the reaction (1). We further consider the formation of iodide Frenkel pairs *V*_I_^+^/*I*_int_^−^, which are frequent sources of iodide defects at surfaces and grain boundaries.^[^ ^43^, ^44^ ^]^ Formation energies of such Frenkel pairs at the FAX‐ and the BuFAX‐terminated surfaces are 0.91 and 0.95 eV, respectively, suggesting only a slight reduction of Frenkel pairs in the presence of BuFA^+^. Notably, both FAX and BuFAX‐terminations strongly passivate defects with respect to the PbX_2_‐terminated case (see Table 1), where significantly lower formation energies of 0.06 eV (*I*_int_^−^/*I*_int_^+^) and 0.30 eV (*V*_I_^+^/*I*_int_^−^) are predicted in line with previous studies.^[^ ^42^, ^43^, ^45^, ^46^ ^]^ At grain boundaries; however, the bulky BuFA cation can reduce the density of PbI_2_‐rich domains, which are key to an enhanced density of iodine Frenkel defects (formation energy of 0.43 eV) in polycrystalline MHP thin‐films.^[^ ^41^ ^]^ Consequently, we may assign the improved phase stability under illumination to a reduced defect density. Moreover, the bulky nature of the cation likely slows down halide migration along surfaces and grain boundaries, further decelerating the iodine release. Still, phase segregation can only be slowed down, while complete suppression is not possible as it remains strongly bound to the iodide‐dominated defect chemistry in lead–halide perovskites, as recently shown.^[^ ^23^, ^42^, ^47^ ^]^

**Table 1:** Defect formation energies at the PbX_2_‐terminated, the FAX‐terminated, and the BuFAX‐terminated surface of the FAPb(*I*_0.67_ *Br*_0.33_)_3_ perovskite. Structural models are visualized in Figure S7, Supporting Information. All energies are given in units of eV.

Defect	PbX2‐term	FAX‐term	BuFAX‐term
*I*_int_^+^/*I*_int_^−^	0.06	0.89	1.20
*I*_2_	0.09	0.20	−0.15
*I*_int_^+^/*I*_int_^−^ → *I*_2_	0.03	−0.69	−1.35
*V*_I_^+^/*I*_int_^−^	0.30	0.91	0.95

**Figure 5:**
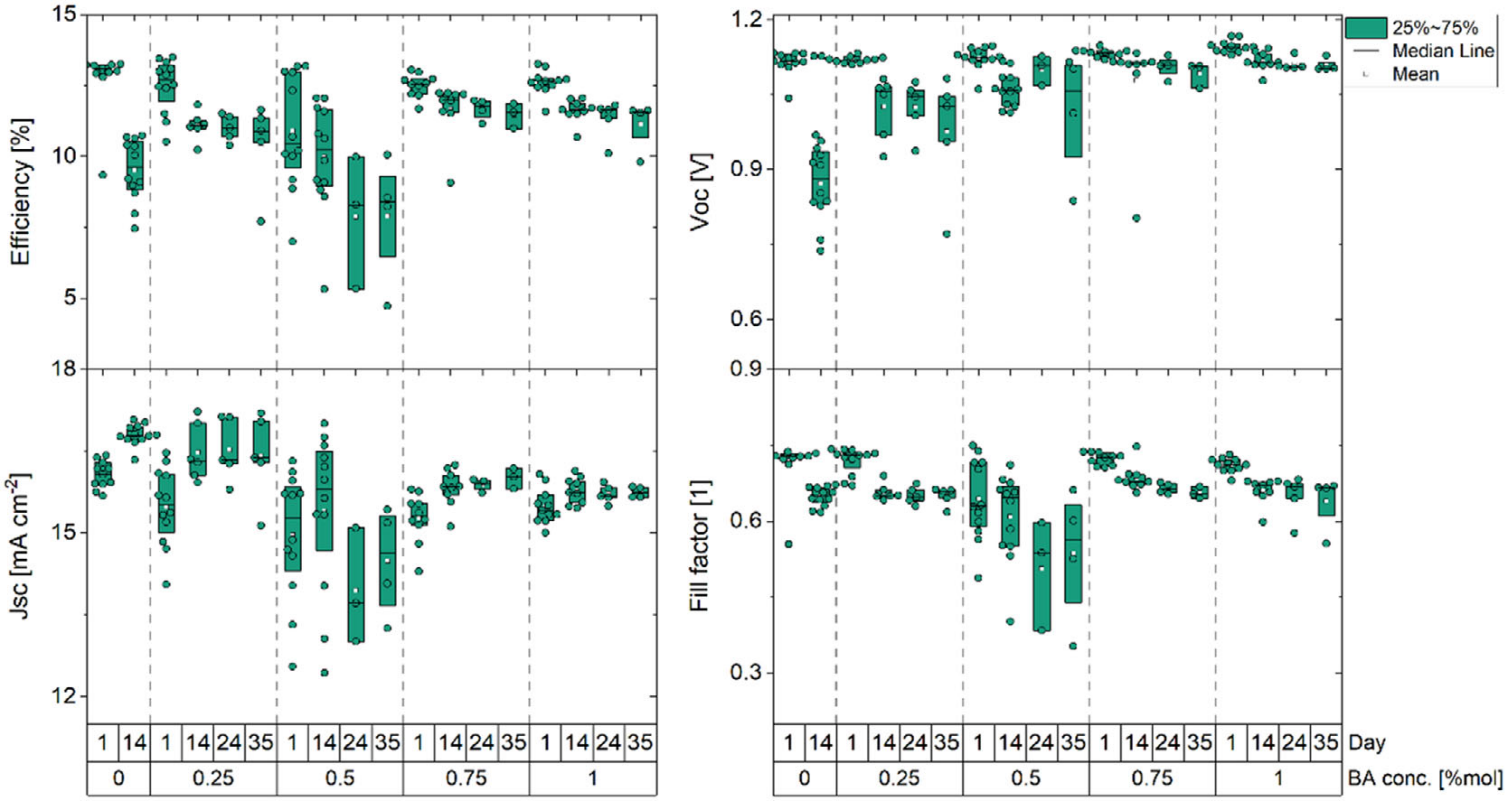
J–V characteristic of samples stored in the glovebox.

**Figure 6:**
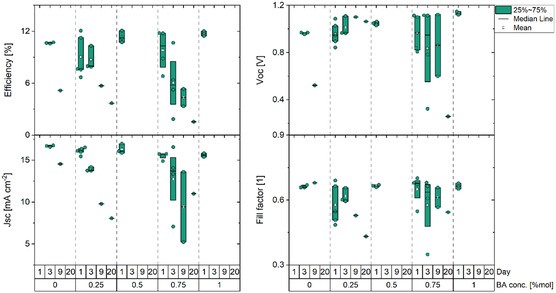
J–V characteristics of samples subjected to continuous illumination in ambient conditions.

Complete perovskite solar cells (PSCs) were produced with the layer stack glass/indium tin oxide (ITO)/PTAA/PFN/WBG perovskite/PCBM/BCP/Al (see Section 4 for more information) (Figure S9a, Supporting Information). These devices were kept in glovebox conditions, under nitrogen for 24 days, and regularly measured under one sun (from Figure S10, Supporting Information, the external quantum efficiency (EQE) spectra used to calibrate the solar simulator to 1 sun intensity can be seen), otherwise they were kept in the dark (**Figure** **5**). Additionally, part of these devices was created with SnO_x_ in place of the BCP, in order to prevent direct contact of the encapsulant with the organic functional layers (BCP, PCBM, and perovskite). These devices were glass–glass encapsulated using an UV curable encapsulant (Figure S9b, Supporting Information). Results from current density–voltage (J–V) measurements, see **Figure** **6** (JV curves for the champion device can be seen in the Figure S11, Supporting Information), come in good agreement with our expectations, as passivated devices show marginal increase in the *V*_OC_. As was also expected, the *J*_SC_ is decreased for increased amounts of BA due to the formation of a low‐dimensional perovskite phase, which is known to hinder current collection.^[^ ^48^ ^]^ For 0.25% mol of BA, *J*_SC_ remains constant as the amount of BA is not sufficient to form 2D phases, as discussed earlier. Interestingly, after 14 days of shelf‐life measurements, the efficiency of the reference devices (denoted with 0 in the additive concentration axis) has decreased more than any passivated device in 24 days. This is a strong finding verifying the increased stability induced by BA addition. Overall, the lowest concentration of BA provides the most uniform and most efficient results by increasing all figures of merit and shows increased shelf‐life stability as well.

Continuous illumination experiments were further performed under one sun illumination in ambient conditions without manipulating the humidity of the atmosphere at 65 °C substrate temperature. It is important to note that the devices were left under open‐circuit conditions at all times, besides during periodic J–V measurements. As shown in Figure 6, on the first day of encapsulation and continuous illumination, all devices are still functional, while after 3 days some devices fail. Until day 20, only the BA‐treated samples with 0.25 mol% BA still operate, reaching 5% efficiency, while maintaining close to 100% of their initial *V*_OC_. It must be noted that the severe *J*_SC_ decrease, besides the degradation of the absorber, can be attributed to the oxidation and decomposition of the electrodes.

## 3 Conclusion

This study provides evidence that the addition of BA in the perovskite absorber can successfully mediate the halide segregation effect, which is an inherent effect in all WBG PSCs. BA reacts with FAI in the liquid perovskite precursor, and the creation of BuFA cations is experimentally proved via ^1^H NMR measurements, which is shown to be favorable by DFT calculations. The bulky BuFA cations cannot be incorporated into the perovskite 3D crystal lattice; thus, leading either to an accumulation at the surfaces and grain boundaries or to the creation of quasi‐2D phases. XRD measurements confirm the formation of quasi‐2D phases at high concentrations (greater than 0.5% mol) of BA, which can impede the absorber's crystallization. Lower BA concentrations improve WBG crystallization and reduce defects, which are key to the instability under illumination. Furthermore, the complete suppression of halide segregation over 15 min under constant illumination of one sun has been demonstrated from extended steady‐state PL measurements at the optimal concentration of 0.25% mol BA. From spatially resolved photoluminescence measurements, a better homogeneity in the emission spectrum of the absorber's surface is shown. Lastly, JV measurements show positive effects on device performance especially for the 0.25% mol BA‐containing devices, showing increased shelf stability and robust behavior under continuous illumination at an elevated temperature of 65 °C. Thus, we conclude that the transamination of the FA cation with tailored concentrations of 0.25% mol BA can effectively produce more reliable PSCs, paving the way for more stable all‐perovskite tandem applications.

## 4 Experimental Section

4.1

4.1.1

##### Methods: Materials

Formamidinium iodide (FAI, 99.99%) was bought from Greatcell Solar Materials. C_60_, lead iodide (PbI_2_, 99.99%) and lead bromide (PbBr_2_, >98%) were bought from TCI chemicals. Butylamine (BA, 99.5%), poly[bis‐(4‐phenyl)‐(2,4,6‐trimethyphenyl)amine] (PTAA), chlorobenzene (anhydrous, 99.8%), N,N‐dimethylformamide (DMF, anhydrous, 99.8%), and dimethyl sulfoxide (DMSO, anhydrous, ≥99.9%), bathocuproine (BCP, 99.99%), and cesium iodide (CsI, 99.99%) were bought from Sigma‐Aldrich. PFN‐P2 was bought from 1‐material.

##### Methods: Perovskite Precursor Solution

WBG perovskite precursor was produced with the stoichiometry FA_0.83_Cs_0.17_Pb(*I*_0.6_ *Br*_0.4_)_3_ with 1.2 M molarity. The perovskite precursor was mixed in DMF:DMSO with 4:1 ratio and stirred at 40 °C for 2 h. After cooling down, varying amounts of BA were added to it. Then, it was filtered with a 0.22 μm polytetrafluoroethylene membrane.

##### Methods: Sample Fabrication

The prepatterned ITO substrates were cleaned in an ultrasonic bath in acetone, isopropanol, and water for 10 min each. After drying, they were moved to a UVO_3_ treatment for 20 min and then in the glovebox for further processing. After that 80 μl of PTAA was dynamically spin‐coated on the substrates at 6000 rpm for 20 s. Then 30 μl of PFN was dynamically spin‐coated on the substrates at 8000 rpm for 30 s, after which they were annealed for 10 min at 100 °C. The perovskite films were created with a two‐step spin‐coating procedure: 1) 2000 rpm with a ramp of 1000 rpm s^−1^ and (2) 4000 rpm for 40 s with a ramp of 2000 rpm s^−1^. The chlorobenzene of 200 μl were dropped on the substrate, 20 s before the end of the second step. Then the films were annealed at 100 °C for 15 min. Later, 20 nm of C_60_ was thermally evaporated, and then 80 μl of BCP was dynamically spin‐coated with 4000 rpm at 1000 rpm s^−1^ for 60 s. Finally, 100 nm of aluminum was thermally evaporated. For the constant illumination measurements, the BCP layer was substituted by a 20 nm atomic layer deposition (ALD) SnO_x_ layer. The processing happened at low temperatures (in the range of 100 °C), using as precursors tetrakis(dimethylamino) tin (IV) (99.9999%, Nanjing Ai Mou Yuan Scientific Equipment Co., Ltd) and deionized water. Lastly, for perovskite films on glass, all hole and electron transport layers were omitted, leaving only the processing of the perovskite precursor on bare glass.

## Conflict of Interest

The authors declare no conflict of interest.

## Author Contributions

**Georgios Loukeris**: conceptualization (lead); data curation (lead); formal analysis (lead); investigation (lead); methodology (lead); visualization (lead); writing—original draft (lead). **Clemens Baretzky**: conceptualization (supporting); investigation (supporting). **Dmitry Bogachuk**: conceptualization (supporting). **Audrey Elizabeth Gillen**: data curation (supporting); investigation (supporting). **Bowen Yang**: data curation (supporting); investigation (supporting); software (supporting). **Jiajia Suo**: data curation (supporting); investigation (supporting); software (supporting). **Waldemar Kaiser**: conceptualization (supporting); investigation (supporting); methodology (supporting); software (lead); writing—original draft (supporting); writing—review and editing (supporting). **Edoardo Mosconi**: investigation (supporting); software (supporting). **Filippo De Angelis**: validation (supporting); writing—review and editing (supporting). **Gerrit Boschloo**: resources (supporting). **Andreas Walter Bett**: resources (supporting); supervision (supporting); writing—review and editing (supporting). **Uli Würfel**: conceptualization (supporting); funding acquisition (supporting); resources (equal); supervision (equal); validation (equal); writing—review and editing (equal). **Markus Kohlstädt**: conceptualization (supporting); funding acquisition (equal); resources (equal); supervision (equal); validation (equal); writing—review and editing (equal).

## Supporting information

Supplementary Material

## Data Availability

The data that support the findings of this study are available from the corresponding author upon reasonable request.
