# Supplementary material for: Suppressing Halide Segregation in Wide‐Bandgap Perovskite Absorbers by Transamination of Formamidinium
Source: Chemphyschem. 2025 Apr 17;26(15):e202500022. doi: 10.1002/cphc.202500022 (PMC12321281; doi:10.1002/cphc.202500022)
Supplement: Supplementary file 1 — Supplementary Material [file CPHC-26-e202500022-s001.pdf]

Supplementary Information for

Suppressing Halide Segregation in Wide-Bandgap Perovskite Absorbers by Transamination of Formamidinium

Georgios Loukeris[*1,2,3], Clemens Baretzky[1,2], Dmitry Bogachuk[1§], Audrey E. Gillen[1,2], Bowen Yang[4], Jiajia Suo[4], Waldemar Kaiser[5†], Edoardo Mosconi[5], Filippo De Angelis[6,7], Gerrit Boschloo[3], Andreas W. Bett[1,3], Uli Würfel[1,2] and Markus Kohlstädt[1,2]

1 Fraunhofer Institute für Solar Energy Systems ISE, 79110 Freiburg im Breisgau, Germany

2 Materials Research Center FMF, University of Freiburg, Freiburg im Breisgau, Germany

3 Institute of Physics, University of Freiburg, Freiburg im Breisgau, Germany

4 Department of Chemistry - Ångström Laboratory, Uppsala, Sweden

5 Computational Laboratory for Hybrid/Organic Photovoltaics (CLHYO), Instituto CNR di Scienze e Tecnologie Chimiche "Giulio Natta" (CNR-SCITEC), 06123 Perugia, Italy

6 Department of Chemistry, Biology and Biotechnology, University of Perugia and INSTM, 06123, Italy

7 SKKU Institute of Energy Science and Technology (SIEST) Sungkyunkwan University, Suwon, Korea 440-746

Email: georgios.loukeris@ise.fraunhofer.de

§ Current address: Solarlab Aiko Europe GmbH, Berliner Allee 29, 79110 Freiburg, Germany.

† Current address: Department of Physics, TUM School of Natural Sciences, Technical University of Munich, 85748 Garching, Germany.

Device Testing

JV measurements

The current density-voltage (*J-V*) characteristics were measured using a Keithley 2400 source meter under illumination from a solar simulator (Newport, Class AAA) with a light intensity of 100 mW/cm^2 (checked with a calibrated reference solar cell from CalLab PV Cells / PV Modules - Fraunhofer ISE). *J-V* curves were measured in a nitrogen atmosphere with a scanning rate of 100 mV/s (voltage step of 10 mV and delay time of 100 ms).

Constant illumination measurements

Devices were put into holder active side facing up into a home-adapted ageing box. The initial model of box is STF-4 and was purchased from Solaronix SA. The lamp used as light source was bought from Plasma International GmbH, and the molen is PLASMA SUUN FF 1450 R. The temperature was controlled with varying fan speeds to keep the air temperature in the chamber at 40 °C. The temperature on the back side of the glass encapsulation was measured with a thermocouple Greisinger GTH 1170 Digital Quick Response Thermometer to be between 50 and 68 °C. Due to the thermal conductivity of glass, there can be an expected temperature gradient through the encapsulation glass thickness. Hence, the perovskite active layer can be significantly higher than the 50 and 68 °C range.

Scanning electron microscopy (SEM)

High-resolution SEM images were obtained using the Hitachi SU7000 microscope with an accelerating voltage of 3 kV. A low accelerating voltage and a low beam current were deployed to reduce surface damage of perovskite films under electron beam bombardment. The substrate structure of our samples for SEM is Glass/ITO/PTAA/PFN/Perovskite.

^{1}H NMR measurements

^{1}H NMR measurements were performed on Bruker AvanceIII-400 MHz NMR spectrometer

XPS measurements

XPS measurements were carried out on an Axis Supra apparatus (Kratos Analytical) using the monochromated Kα X-ray line of an aluminum anode.

XRD measurements

The X-ray diffraction patterns were recorded with PANalytical Empyrean system with a PIXcel-1D detector, Bragg-Brentano beam optics and parallel beam optics. Light source is from copper Kα beam filtered with nickel β filter. Diffraction spectra were characterized between 2-theta of 5° and 50° at a scan rate of 1° per minute with the step width of 0.02^{o}

Photoluminescence (PL) characterization

The excitation source was a focused beam of a 532 nm continuous diode laser. The PL spectra were collected using an Andor Solis software system. To measure the photostability, the perovskite films were illuminated under 1 sun (AM 1.5 G) and the illumination impinged on the glass side. For the extended PL spectra of the films no encapsulation was conducted.

Quasi-Fermi level splitting (QFLS) characterizations

Perovskite films were deposited on glass with the same recipe used for device fabrication. No encapsulation of the samples was conducted before the measurement. The absolute PL spectra of the samples were collected using the LuQY Pro (HZB). Samples were excited by a 532 nm laser at 1 sun illumination condition.

Transient photoluminescence (tr-PL) characterization

Perovskite films deposited on glass substrates were subjugated to 1 sun illumination from a pusled focus laser beam of 532 nm. No encapsulation of the samples was conducted before the

measurement. To identify the 1 sun intensity, the photocurrent of complete PSCs was measured in the setup. The data collected were analysed using the software origin Pro.

Micro photoluminescence (µ-PL) characterization

Perovskite films deposited on glass substrates were subjugated to illumination from a continuous focused laser beam of 532 nm. No encapsulation of the samples was conducted before the measurement. The WITec control and WITec software were used for data acquisition and data analysis, as well as controlling the XYZ stage where the samples were located.

Computational details

Density functional theory (DFT) calculations have been carried out within the Quantum Espresso software package [1]. We made use of the PBE [2] as exchange-correlation potential and approximated the core regions with ultrasoft pseudopotentials (valence shells explicitly included in calculations: Br 4s, 4p; I 5s, 5p; N, C 2s, 2p; H 1s; Pb 6s, 6p, 5d). A plane-wave cutoff for the wavefunctions of 40 Ryd (320 Ryd on the charge density) was used. DFT-D3 correction were also included in the geometry optimization [3].

To model the mixed-halide perovskite $FAPb(I_{0.67}Br_{0.33})_3$, we replaced homogeneously I^- by Br^- anions within a 2×2×2 supercell of the β-phase of $FAPbI_3$ [4], followed by a geometry relaxation with flexible cell boundaries. The resulting supercell is of dimensions a = 17.81629 Å, b = 17.77180 Å, c = 24.86917 Å with angles α = 89.87571°, β = 90.02664°, and γ = 89.69238°. Subsequently, slab models have been built with different terminations (PbI_2 and FAX), containing of 5 inorganic layers. Additionally, a minimum of 20 Å vacuum was added along the non-periodic direction perpendicular to the slabs in all cases to avoid spurious interactions between the periodic replicas. To model the BuFAX-terminated slab, the FA^+ cations from the

FAX-terminated slab have been replaced by $BuFA^+$ cations followed by a geometry relaxation of the surface models.

The formation energy of the I_i^+/I_i^- defect pair at the surface has been modeled in analogy to [5] by:

$$E_F(\mathrm{I}_{\mathrm{int}}^{+}/\mathrm{I}_{\mathrm{int}}^{-}) = E(\mathrm{def.}) - E(\mathrm{prist.}) - 2\mu(I) \quad (1)$$

where $E(\mathrm{prist.})$ is the pristine reference system, $E(\mathrm{def.})$ is the respective system with the I_i^+/I_i^- defect pair, and μ(I) is the chemical potential of an iodine atom obtained from the geometry optimized solid I_2 phase, COD ID 9008595 [6]. Defect formation energies of iodide Frenkel pairs $\mathrm{V}_{\mathrm{I}}^{+}/\mathrm{I}_{\mathrm{int}}^{-}$ are obtained as

$$E_F(\mathrm{V}_{\mathrm{I}}^{+}/\mathrm{I}_{\mathrm{int}}^{-}) = E(\mathrm{def.}) - E(\mathrm{prist.}) \quad (2)$$

Note that, as all considered defects are charge neutral, no electrostatic corrections are required.

DFT calculations of the reaction of the BA molecule with the FA^+ cation have been carried out using Gaussian09 program package [Frisch, M. J.; Trucks, G. W.; Schlegel, H. B.; Scuseria, G. E.; Robb, M. A.; Cheeseman, J. R.; Scalmani, G.; Barone, V.; Petersson, G. A.; Nakatsuji, H.; et al. Gaussian 09, Revision D. 01. Gaussian, Inc. Wallingford CT 2016] with the hybrid B3LYP exchange-correlation functional [7] along with the 6-311G** basis set for the all chemical species, and the implicit solvation model C-PCM [10.1002/jcc.10189] with dimethyl-sulfoxide was chosen to account for the solvent environment in the simulation.

Supplementary figures

Full ^{1}H NMR spectra

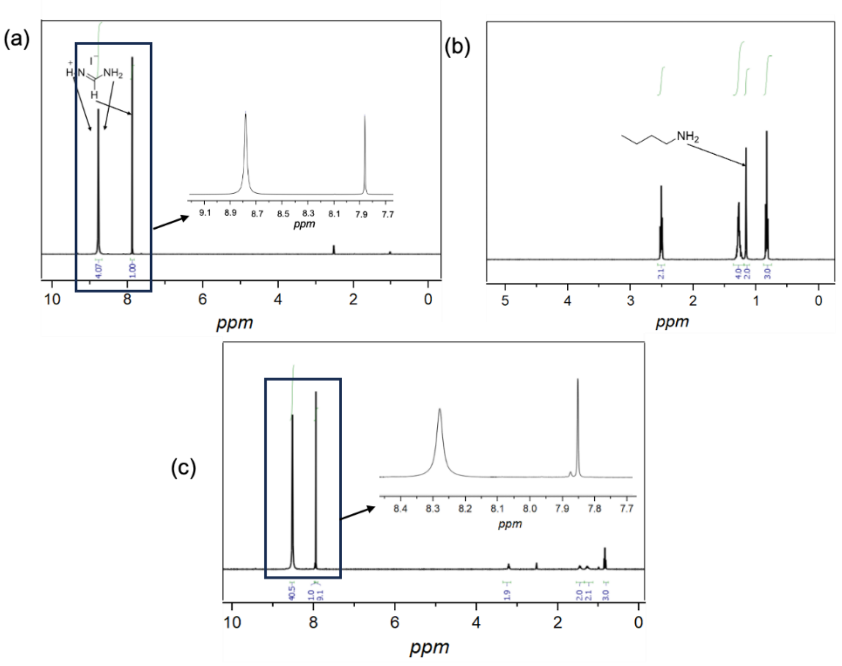

Supplementary Figure 1. (a) ^{1}H NMR spectra of FAI over the full measuring range (inset shows the FAI spectra). (b) ^{1}H NMR BA spectra (the green lines correspond to the peaks). (b) ^{1}H NMR FAI+BA spectra. The highlighted regime of BA+FAI interaction is shown in the inset.

Full XPS spectra

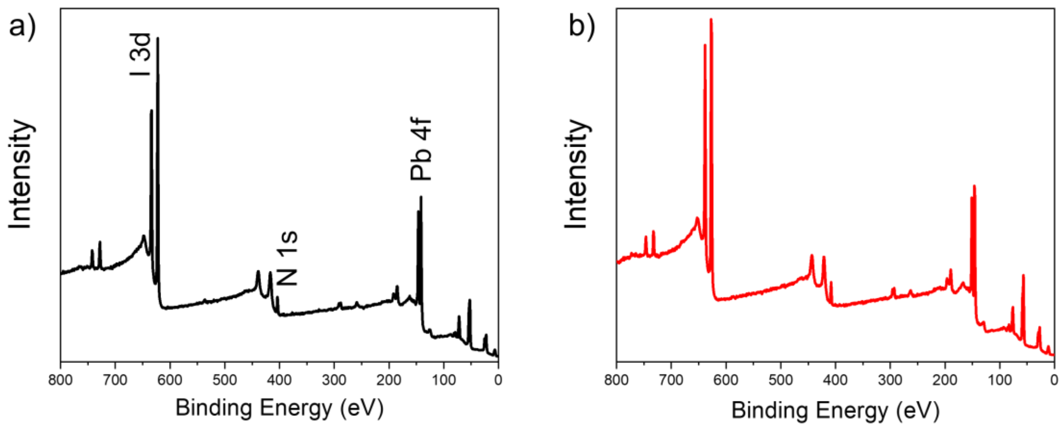

Supplementary Figure 2. Full XPS spectra of (a) undoped and (b) BA doped perovskite film on glass.

Micro-PL (µPL)

Images of thin films containing concentration of BA can be seen in the figure bellow.

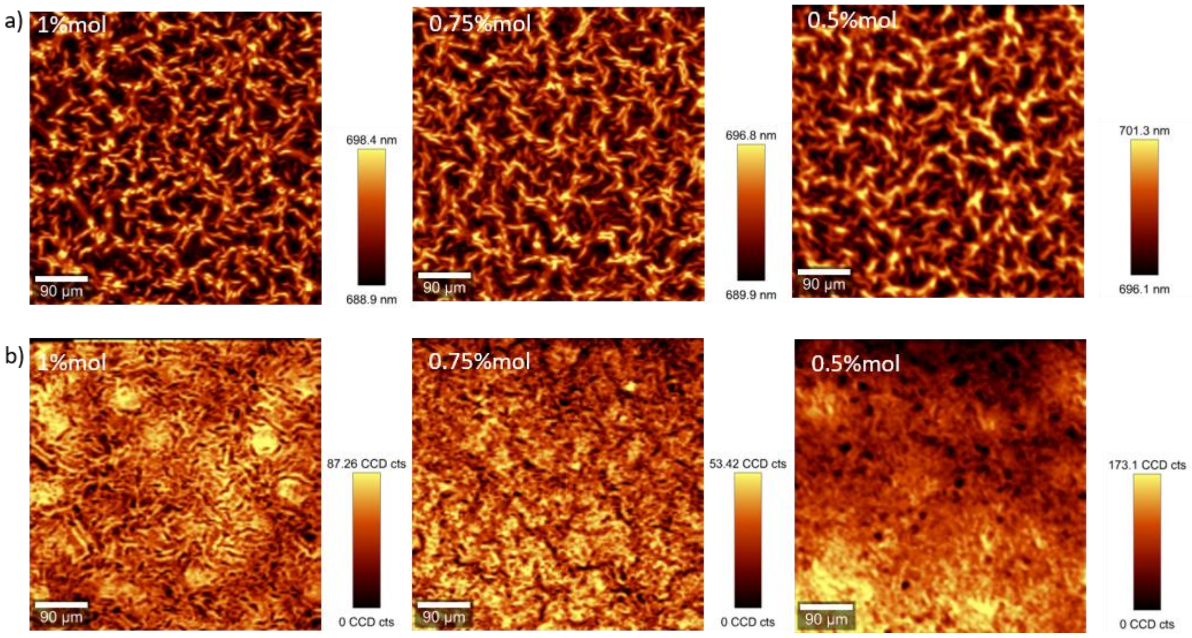

Supplementary Figure 3. µPL spectra of varying the BA concentration. a) Spectrally resolved and b) Intensity resolved.

Optical Microscopy

Optical Microscopy images were taken from BA-treated perovskite films of glass substrate.

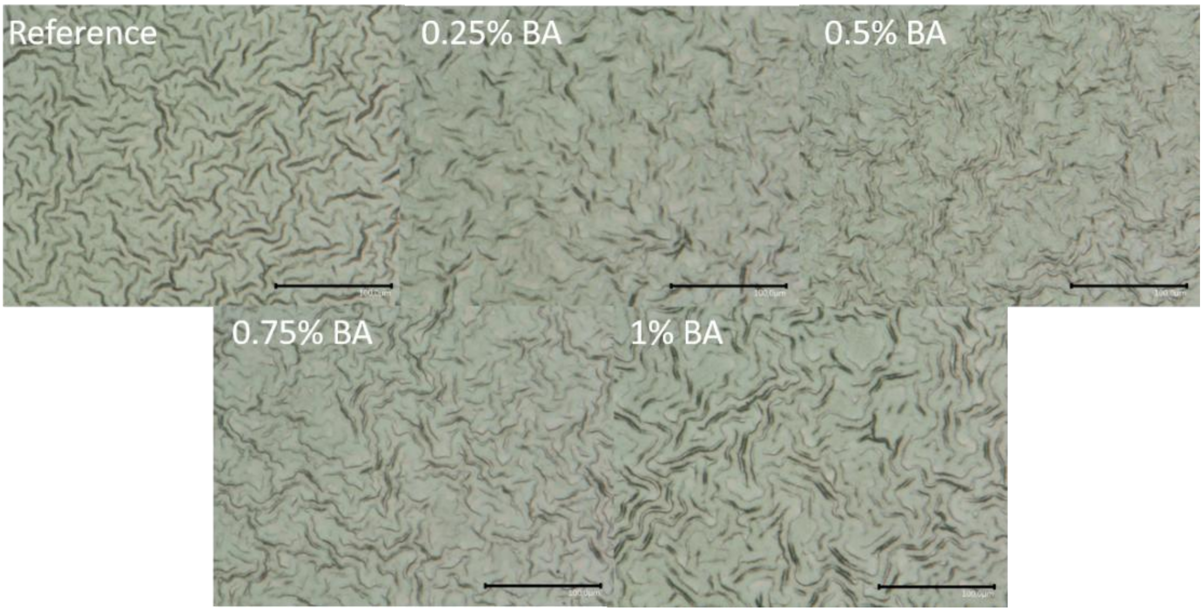

Supplementary Figure 4. Microscopy images of WBG perovskite films with varying BA concentration. The scale bar corresponds to 100 µm.

Absorption-reflection-transmission (RTA)

RTA spectra of reference and BA-treat perovskite films on glass substrate.

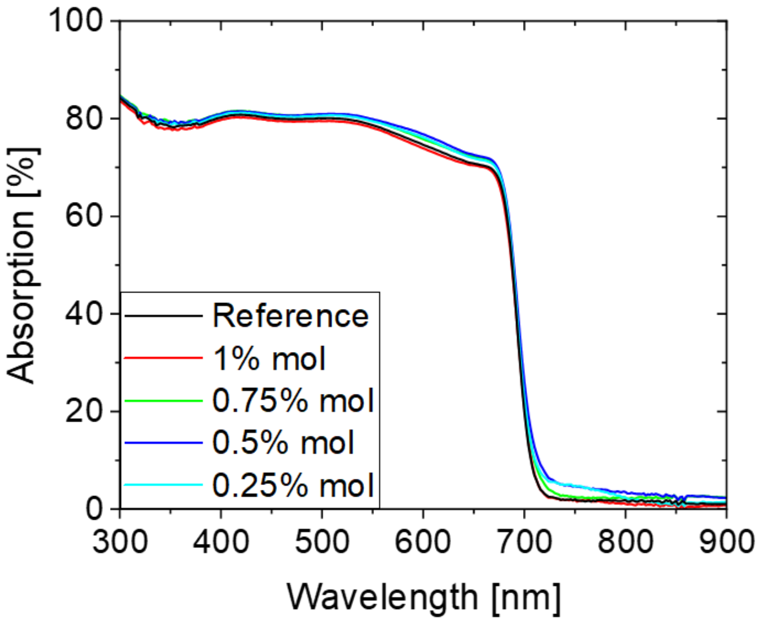

Supplementary Figure 5. RTA spectra of reference and BA-treated perovskite films.

Steady state PL

Data for all Butylamine concentration variations performed using the same setup as mentioned in the main text.

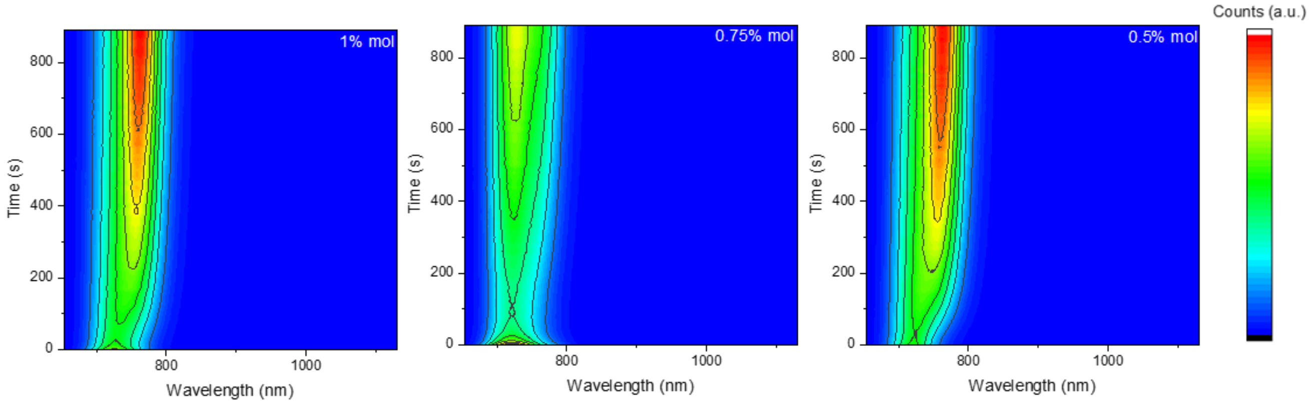

Supplementary Figure 6. PL images for varying BA concentrations.

Transient photoluminescence (tr-PL)

Tr-PL spectra of reference and BA-treat perovskite films on glass substrate.

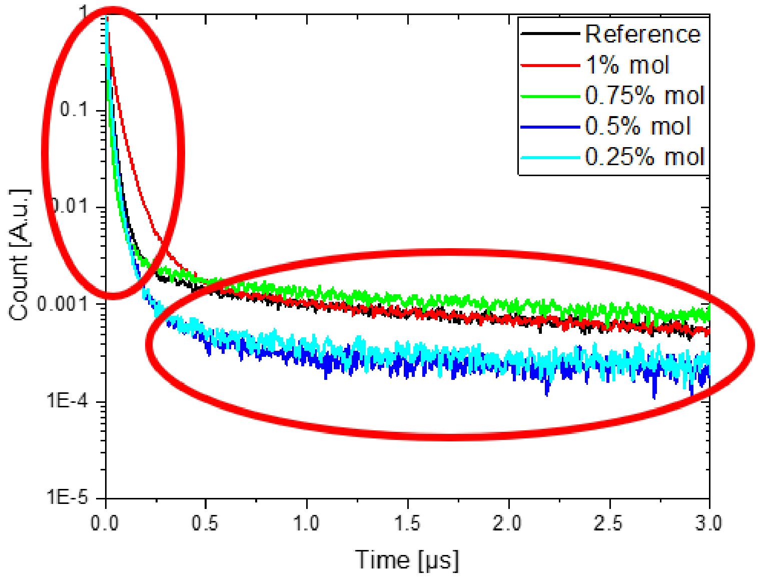

Supplementary Figure 7. Transient photoluminescent spectra of reference and BA-treated perovskite films. With the red circles decay areas of interest are enclosed.

Perovskite slab models

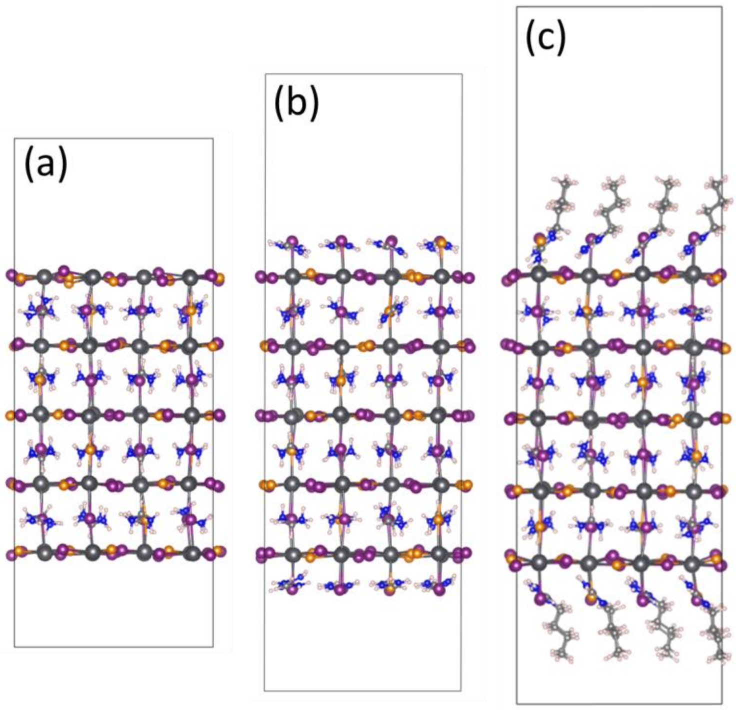

Supplementary Figure 8. Slab models of the $FAPb(I_{0.67}Br_{0.33})_3$ models with (a) PbX_2-termination, (b) FAX-termination, and (c) BuFAX-termination.

Layer stacks used for measuring the solar cell performance

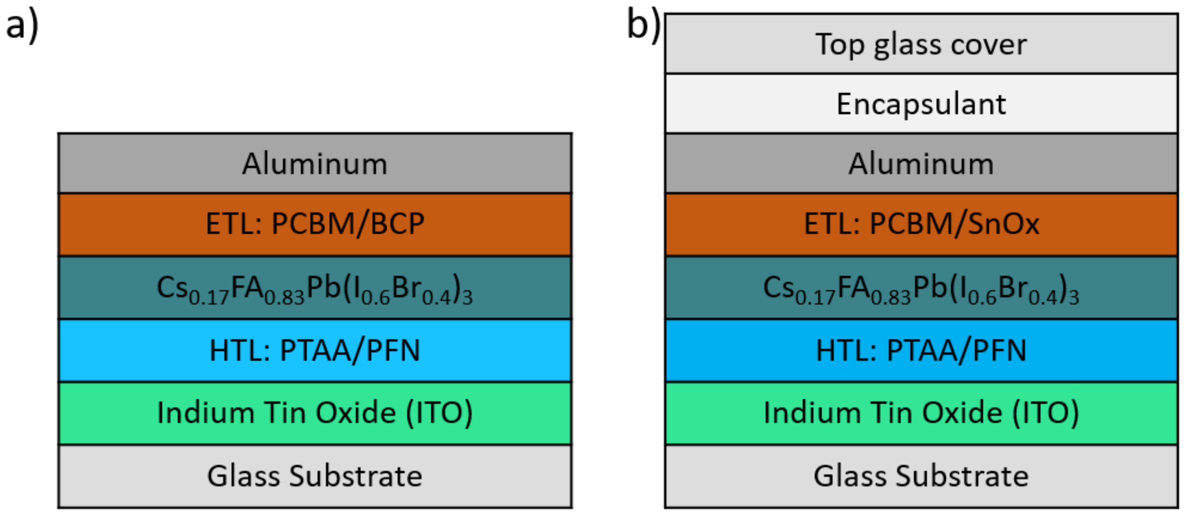

Supplementary Figure 9. Device layout used for single jhunction perovskite solar cells. a) Layer stack used for the long-term shelf-life stability experiments. b) Layer stack used for the continuous illumination experiments.

EQE spectra of the WBG perovskite absorber

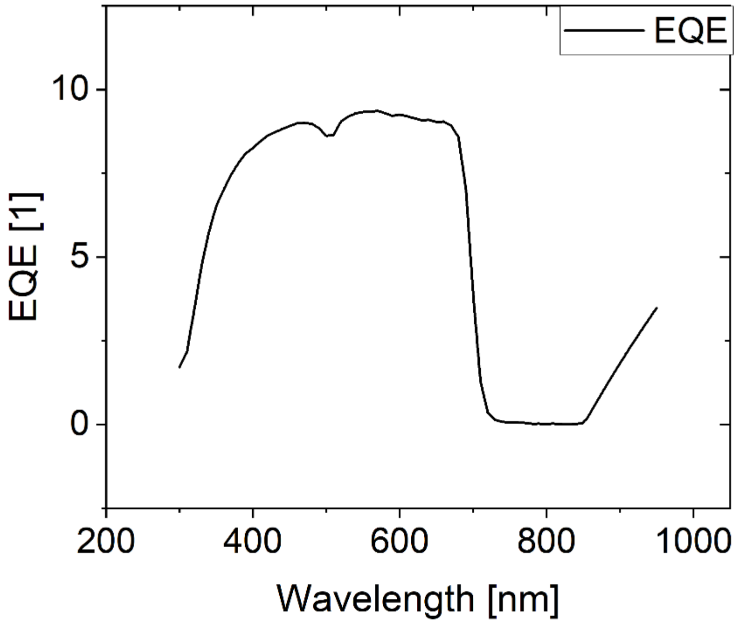

Supplementary Figure 10. EQE spectra of the WBG absorber. Note that this is a relative measurement. The spectra are collected in order to identify the band gap of the absorber and to set the solar simulator at 1sun intensity for appropriate measuring.

JV curves of champion device and corresponding data

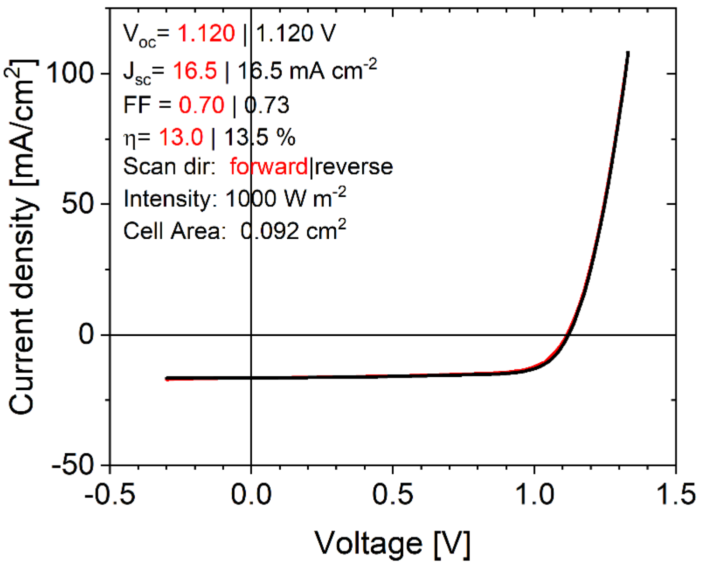

Supplementary Figure 11. Forward (red) and reverse black) biasing JV curves of the champion device corresponding to 0.25% mol BA.

References

[1] P. Giannozzi *et al.,* "QUANTUM ESPRESSO: a modular and open-source software project for quantum simulations of materials," *Journal of physics. Condensed matter : an Institute of Physics journal*, vol. 21, no. 39, p. 395502, 2009, doi: 10.1088/0953-8984/21/39/395502.

[2] Perdew, Burke, and Ernzerhof, "Generalized Gradient Approximation Made Simple," *Phys. Rev. Lett.*, vol. 77, no. 18, pp. 3865–3868, 1996, doi: 10.1103/PhysRevLett.77.3865.

[3] S. Grimme, J. Antony, S. Ehrlich, and H. Krieg, "A consistent and accurate ab initio parametrization of density functional dispersion correction (DFT-D) for the 94 elements H-Pu," *J. Chem. Phys.*, vol. 132, no. 15, p. 154104, 2010, doi: 10.1063/1.3382344.

[4] Aron Walsh, elds22, Federico Brivio, and Jarvist Moore Frost, *WMD-group/hybrid-perovskites: Collection 1*: Zenodo, 2019.

[5] S. Martani *et al.,* "Defect Engineering to Achieve Photostable Wide Bandgap Metal Halide Perovskites," *ACS Energy Lett.*, vol. 8, no. 6, pp. 2801–2808, 2023, doi: 10.1021/acsenergylett.3c00610.

[6] S. Gražulis *et al.,* "Crystallography Open Database - an open-access collection of crystal structures," *Journal of applied crystallography*, vol. 42, Pt 4, pp. 726–729, 2009, doi: 10.1107/S0021889809016690.

[7] A. D. Becke, "Density-functional thermochemistry. III. The role of exact exchange," *J. Chem. Phys.*, vol. 98, no. 7, pp. 5648–5652, 1993, doi: 10.1063/1.464913.
